# Emerging Applications of Nanoparticles in the Diagnosis and Treatment of Breast Cancer

**DOI:** 10.3390/jpm14070723

**Published:** 2024-07-04

**Authors:** Josephine B. Oehler, Weranga Rajapaksha, Hugo Albrecht

**Affiliations:** 1 College of Medicine and Dentistry, James Cook University, Townsville, QLD 4810, Australia; 2 Biomedical Sciences and Molecular Biology, College of Public Health, Medical & Vet Sciences, James Cook University, Townsville, QLD 4810, Australia; 3 Centre for Pharmaceutical Innovation (CPI), Clinical and Health Sciences, University of South Australia, Adelaide, SA 5000, Australia

**Keywords:** breast cancer, tumor, biomarkers, diagnostics, therapeutics, theragnostics, theranostics, nanomedicine

## Abstract

Breast cancer remains the most prevalent cancer among women worldwide, driving the urgent need for innovative approaches to diagnosis and treatment. This review highlights the pivotal role of nanoparticles in revolutionizing breast cancer management through advancements of interconnected approaches including targeted therapy, imaging, and personalized medicine. Nanoparticles, with their unique physicochemical properties, have shown significant promise in addressing current treatment limitations such as drug resistance and nonspecific systemic distribution. Applications range from enhancing drug delivery systems for targeted and sustained release to developing innovative diagnostic tools for early and precise detection of metastases. Moreover, the integration of nanoparticles into photothermal therapy and their synergistic use with existing treatments, such as immunotherapy, illustrate their transformative potential in cancer care. However, the journey towards clinical adoption is fraught with challenges, including the chemical feasibility, biodistribution, efficacy, safety concerns, scalability, and regulatory hurdles. This review delves into the current state of nanoparticle research, their applications in breast cancer therapy and diagnosis, and the obstacles that must be overcome for clinical integration.

## 1. Introduction and Background

Breast cancer stands as a formidable global health challenge, with approximately 2.3 million new cases diagnosed annually [1]. Its multifaceted nature, characterized by diverse molecular subtypes and clinical presentations, necessitates personalized approaches to management. Genetic anomalies, notably in BRCA1 and BRCA2 genes, contribute significantly to disease predisposition, while molecular pathways such as PI3K/AKT/mTOR drive tumorigenesis [2].

Contemporary treatment models, integrating surgery, cytotoxic, hormonal, and targeted therapies, are tailored to the tumor’s molecular profile and clinical stage. However, oncologists are faced with significant challenges when managing breast cancer, including high recurrence rates, therapeutic resistance, metastatic dissemination, and treatment toxicities [3,4].

Nanoparticles, with sizes ranging from 10 to 100 nanometers, show immense promise in the treatment of breast cancer [5]. Due to their minute size, these particles have a unique set of physical and chemical properties including a large reactive surface area relative to their volume and a chemical makeup that can be easily modified. Hence, nanoparticles can be used to carry therapeutic drugs directly to cancer cells, potentially increasing treatment efficacy while minimizing harm to healthy cells [6]. The combination of nanoparticle movement across the body’s barriers with the tendency to accumulate in tumor tissues makes them a powerful tool for both detecting and treating cancer more effectively [7]. Moreover, the integration of multi-omics approaches, which include genomics, proteomics, and metabolomics, is transforming the understanding and treatment of breast cancer. These approaches provide a comprehensive view of the molecular underpinnings of cancer, enabling better targeted therapies. For instance, combining nanoparticles with multi-omics data can enhance the identification of biomarkers and improve the delivery of targeted therapies.

Indeed, the utilization of nanoparticles in breast cancer therapy has garnered significant attention owing to their potential to optimize drug delivery and therapeutic outcomes. By expanding the therapeutic window—the range of drug dosages that are effective yet non-toxic—nanoparticles mitigate the risk of adverse effects while ensuring that optimal drug concentrations will reach cancerous cells. Furthermore, selective targeting of cancer cells holds promise for minimizing off-target effects, a critical consideration in current cancer treatments [7].

## 2. Classification of Nanoparticles

Nanoparticles, defined by their dimensions within the nanoscale (under 100 nm), exhibit distinct properties setting them apart from conventional materials [8]. Their small size provides unique characteristics, including enhanced chemical reactivity, heightened energy absorption capabilities, and increased biological mobility. Table 1 outlines the diverse landscape of nanoparticle classifications and their corresponding physicochemical characteristics.

The engineering of nanoparticles involves a meticulous consideration of particle size, surface charge, surface functionality, and drug loading capacity [3]. By conjugating nanoparticles with targeting ligands—such as antibodies, peptides, or small molecules—active targeting is enhanced. The intrinsic properties of each nanoparticle class can be harnessed for specific therapeutic purposes. For instance, lipid-based nanoparticles, such as liposomes and lipid nanoparticles, offer biocompatible carriers for drug delivery, facilitating controlled release and the co-delivery of multiple agents [9]. Polymeric nanoparticles, composed of materials like poly(lactic-co-glycolic acid) (PLGA) and polyethylene glycol (PEG) afford customizable platforms for gene therapy and targeted drug delivery [19,20]. Inorganic nanoparticles, including gold and iron oxide, leverage unique properties for polythermal therapy (PTT) and imaging [21,22]. Protein-based nanoparticles, such as albumin and ferritin, harness natural ligands for targeted drug delivery with reduced immunogenicity [15]. Carbon-based nanoparticles, such as carbon nanotubes and graphene, demonstrate promise in PTT and bioimaging [22]. Hybrid nanoparticles combine diverse materials to synergistically enhance the therapeutic efficacy and imaging capabilities [8,17]. Gold nanoparticles (AuNPs) have garnered significant attention due to their unique physicochemical properties, including facile modulation of shape and size, high degree of reproducibility and stability, and biocompatibility [23]. These properties make AuNPs particularly valuable in breast cancer applications such as PTT, radiotherapy, molecular labelling, imaging, and sensing [23].

Their high atomic number and surface plasmon oscillation are particularly advantageous for theranostic applications, allowing for both therapeutic and diagnostic capabilities in a single platform. The optical properties of AuNPs, such as the ability to absorb and scatter light, are exploited in PTT to induce localized hyperthermia, effectively destroying cancer cells while sparing healthy tissue [24,25]. Moreover, the integration of gold nanorods and black phosphorus in optical microfiber sensors has shown high sensitivity in detecting and targeting breast cancer biomarkers, enabling early diagnosis and targeted treatment [26].

Despite these promising attributes, the clinical translation of AuNPs faces significant hurdles. Toxicity in major organs such as the liver, kidneys, and spleen remain a primary concern, necessitating ongoing research into safer particle synthesis and engineering techniques to enhance efficacy while minimizing adverse effects [23,27]. This challenge underscores the importance of developing strategies that can improve the safety profile of AuNPs, ensuring their feasible application in clinical settings [27].

Recent advances in inorganic nanoparticles (INPs), including metal, metal oxide, and noble metal nanoparticles, have further broadened the therapeutic landscape. INPs such as iron oxide, silver, and silica nanoparticles are being explored for their unique magnetic, thermal, and catalytic properties, which enhance their functionality in imaging, drug delivery, and therapeutic applications [22,28]. For instance, iron oxide nanoparticles (IONPs) are particularly promising due to their magnetic properties, which can be utilized in magnetic resonance imaging (MRI) and for targeted drug delivery through magnetic targeting.

Additionally, INPs can be engineered to possess multifunctional capabilities. For example, silica nanoparticles can be modified to carry therapeutic agents while also serving as imaging contrast agents. This dual functionality allows for simultaneous therapy and diagnostic imaging, enhancing the precision of cancer treatment (theranostics) [22]. Such integrated approaches facilitate real-time monitoring of treatment efficacy and adjustment of therapeutic strategies, thus optimizing patient outcomes. These examples underscore the huge potential of nanoparticle-based strategies in the ongoing battle against breast cancer [29].

## 3. Diagnostic Potential of Nanoparticles in Breast Cancer

**Figure 1:**
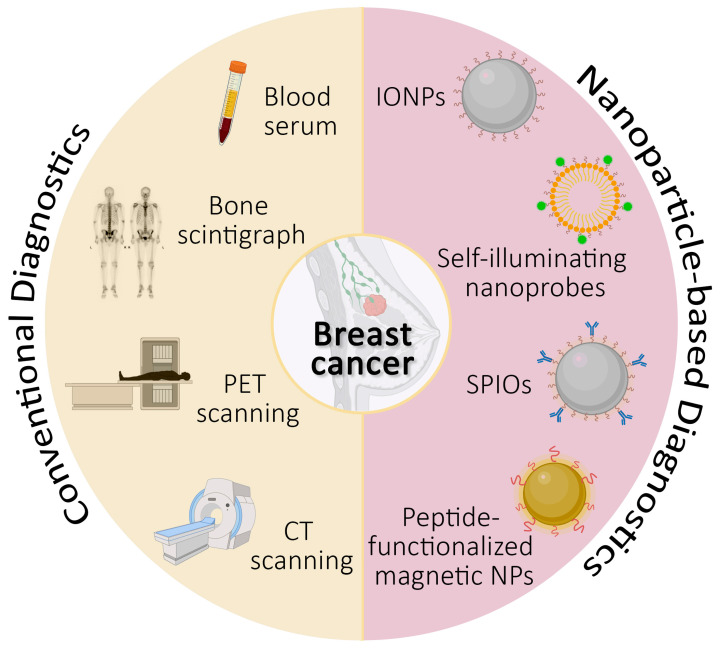
Diagram of conventional and nanoparticle-based approaches in breast cancer diagnosis. IONPs: Iron oxide nanoparticles, SPIOs: superparamagnetic iron oxide nanoparticles, PET: positron emission tomography, CT: computed tomography, NPs: nanoparticles.

The early detection of breast cancer metastasis remains a critical determinant for therapeutic success and patient prognosis. Conventional diagnostic modalities, including blood tests, bone scintigraphy, and positron emission tomography–computed tomography (PET/CT) imaging (Figure 1), are frequently challenged by their limited ability to identify incipient metastatic lesions, especially those at remote sites. Recent progress in nanotechnology has catalyzed a transformative approach to cancer diagnostics [30,31]. Nanoparticles, with their specific surface chemistry and structural versatility that facilitate their preferential accumulation in tumor tissues, have emerged as powerful agents capable of increasing diagnostic sensitivity and specificity (Figure 1) [30,32].

The currently used blood tests and scanning procedures are expected to be enhanced through their combination with novel nanoparticle-based methods, such as the use of iron oxide nanoparticles (IONPs) for imaging of cancer metastasis in the brain [33], self-illuminating nanoprobes as markers for tumor-infiltrating neutrophils [34], ATP-responsive superparamagnetic iron oxide nanoparticles (SPIOs) [35], and peptide-functionalized magnetic nanoparticles to detect human epidermal growth factor receptor 2 (HER2) on circulating tumor cells [36].

This has been exemplified by the study of Du et al., where ultrasmall iron oxide nanoparticles (IONPs) were employed for the precise imaging of breast cancer brain metastasis [33]. The IONPs were modified with a peptide derived from phage display (breast carcinoma cell (231-BR)-binding peptide colloquially known as BRBP1), which displays high binding affinity towards breast cancer cells that spread to the brain [33]. This targeted nanoparticle offered enhanced imaging contrast and specific uptake by tumor tissue, thus improving the detection accuracy for metastasis. A different nanoparticle-based imaging method was presented by Zheng et al., who developed a pioneering nanoprobe for the detection of lung metastasis [34]. By utilizing self-illuminating nanoprobes that target neutrophil infiltration—a critical early indicator of metastasis—their approach detected metastatic breast cancer cells in the lung with a notable 98% sensitivity and 96% specificity [34]. These examples demonstrate the huge potential of nanoparticle-based imaging strategies in the early detection of metastasis, thereby promising to refine the precision of personalized breast cancer treatment strategies [34].

Wang G et al. took the versatility of nanoparticle applications further into the field of dual-modality imaging by pioneering the use of superparamagnetic iron oxide nanoparticles (SPIOs) [35]. These nanoparticles are particularly responsive to ATP and therefore sense high metabolic activity in tumors. When introduced into the body, the nanoparticles—equipped with a targeted peptide—accumulated in metastatic lymph nodes and emitted a strong molecular fluorescence. In addition, this method provided more profound tissue penetration, leading to highly sensitive detection of metastatic disease [35]. Liang et al.’s research using PDT complements this approach by addressing a different stage in cancer management: early detection and precise treatment initiation [37]. For this purpose, an optical microfiber sensor with integrated gold nanorods and black phosphorus was established [37].

In addition to the targeted imaging of metastasis, nanoparticle-based platforms have shown promise in detecting circulating tumor cells (CTCs). The detection and analysis of CTCs provide invaluable insights into the pathophysiology of metastasis and cancer progression [38]. Given that CTCs are a hallmark of metastatic disease, their early identification is critical for the timely initiation of personalized therapeutic regimens and could be instrumental in improving patient prognosis. A good example is the work of Wang M et al., who developed an innovative fluorescent technique using peptide-functionalized magnetic nanoparticles to quantify HER2 on CTCs [36]. This method not only quantifies these cells but also provides prognostic data, potentially guiding therapy choices and thereby improving patient outcomes [36].

A list of the most recent clinical trials with diagnostic nanoparticles for breast cancer is given in Table 2. SPIOs and carbon nanoparticles are the dominant compositions, apart from one quantum dot-based imaging system. Notably, all of these trials aim to locate lymph nodes, with the exception of NCT04138342, which is intended for breast cancer bioimaging in general.

These advancements of nanoparticle-based diagnostics provide unprecedented imaging accuracy and the means to monitor treatment efficacy in real time. However, this innovation does not come without caveats. Potential risks like systemic toxicity and immune reactions stemming from nanoparticle interaction with biological systems call for stringent safety evaluations [39,40]. In particular, attention to nanoparticle interactions with non-target tissues is essential to prevent oxidative stress and other adverse effects and underscore the paramount importance of a measured advancement in nanoparticle diagnostics.

## 4. Therapeutic Potential of Nanoparticles in Breast Cancer

In addition to their diagnostic potential, nanoparticles hold immense promise in revolutionizing breast cancer treatment. Nanoparticles offer unique advantages in the delivery of therapeutics and overcoming chemoresistance, enabling targeted therapy with enhanced efficacy and reduced systemic toxicity [6,41]. Table 3 provides an overview of breast cancer nano-formulations, which have been approved for clinical use or are undergoing clinical trial. In addition to the listed trials, there are currently over 60 albumin-based formulations being tested in the clinic to expand on the strategy successfully established with Abraxane.

A broad range of nanoparticle formulations, as detailed in Table 3, is either currently used in clinical settings or under development, showcasing the potential of these technologies to refine oncological therapeutics through targeted and efficient drug delivery mechanisms.

Abraxane, an albumin-bound formulation of paclitaxel, is a notable example of how nanoparticles can be utilized effectively in cancer therapy [42]. This formulation takes advantage of the natural properties of albumin to passively target tumor tissues through the enhanced permeability and retention (EPR) effect. The EPR effect is crucial for the accumulation of therapeutic agents within the tumor microenvironment, facilitated by the defective vascular architecture and poor lymphatic drainage characteristic of tumor sites. This passive targeting mechanism allows for a higher concentration of the drug to reach the tumor, enhancing its efficacy while reducing the toxic side effects typically associated with systemic distribution [42,43].

Genexol-PM and Nanoxel M, both formulations of polymeric micelle paclitaxel, are also approved for use and employ similar passive targeting mechanisms [44]. These polymeric micelles enhance the solubility and stability of paclitaxel, facilitating its accumulation at the tumor site through the EPR effect. The polymeric structure of these nanoparticles allows for a controlled release of the drug, maintaining therapeutic levels in the plasma for extended periods and reducing the frequency of administration required.

Active targeting strategies are employed by newer nanoparticle formulations like ELU001 and CALAA-01, which are designed to improve the specificity of drug delivery by targeting molecules overexpressed on cancer cells [45]. ELU001 targets folate receptor 1 (FOLR1), a receptor abundantly expressed in certain breast cancer subtypes [46], using C-Dot nanoparticles. This targeting strategy aims to deliver cytotoxic agents directly to cancer cells, maximizing the therapeutic effect while minimizing damage to normal tissues. ELU001 is currently being evaluated in phase I/II clinical trials to assess its safety, tolerability, and preliminary efficacy.

CALAA-01 represents a significant advancement in targeted therapy using nanoparticles to deliver siRNA specifically to cancer cells [47]. By targeting the transferrin receptor, which is upregulated in many cancer patients due to their increased iron requirements, CALAA-01 encapsulates siRNA directed against ribonucleotide reductase M2, a key enzyme in DNA synthesis. This targeted approach not only enhances the drug’s efficacy but also limits its systemic exposure, potentially reducing the adverse effects typically seen with conventional chemotherapy [48]. CALAA-01 is under phase I clinical trials, focusing on its capacity to selectively inhibit tumor growth while sparing healthy cells.

These examples illustrate the dynamic nature of nanoparticle research in oncology, highlighting the significant strides made in enhancing drug delivery systems, improving patient outcomes, and reducing treatment-related toxicities. The development of such targeted therapies exemplifies a shift towards personalized medicine, where treatments are tailored to exploit the unique characteristics of the tumor microenvironment and the molecular profile of the tumor cells.

### 4.1. Passive Targeting

In nanoparticle-mediated drug delivery, passive targeting exploits the unique physiological characteristics of the tumor to deliver therapeutic agents more effectively. Passive targeting via the EPR effect is a common strategy. EPR is a phenomenon observed in tumor tissues where newly formed blood vessels are leaky due to larger fenestrations between endothelial cells than those in normal tissues, allowing nanoparticles to accumulate preferentially in the tumor microenvironment [43]. The EPR effect is significantly enhanced for nanoparticles ranging from 10 to 100 nanometers, which also escape rapid renal clearance, thus increasing the therapeutic payload delivered to the tumor while minimizing systemic toxicity [43,49,50].

Doxil^®^, the first FDA-approved nanoparticle drug, consists of liposomal doxorubicin designed to exploit the EPR effect for targeted drug delivery in breast cancer. Clinical trials demonstrated improved patient outcomes with Doxil^®^ by reducing the cardiotoxicity associated with free doxorubicin while maintaining its anticancer efficacy [51].

Recent advancements in the field have seen the development of more sophisticated nanoparticle systems that enhance the EPR effect. For example, the surface modification of nanoparticles with hydrophilic polymers such as PEG has been shown to reduce protein adsorption, thereby prolonging circulation time and enhancing tumor accumulation through the EPR effect. This strategy was effectively employed in the formulation of Abraxane^®^, which exhibits increased tumor accumulation and has been approved for treating metastatic breast cancer [42,52].

More recently, tumor pH, hypoxia, and enzymatic activity have been identified as key modifiers of nanoparticle behavior in the tumor milieu affecting the EPR effect. Chen et al., highlighted that the acidic and hypoxic conditions of the tumor microenvironment can be exploited to design pH-sensitive nanoparticles that release their payload in response to low pH, enhancing drug release directly at the tumor site, thereby improving therapeutic outcomes [53]. Furthermore, ongoing research continues to refine the design of nanoparticles to overcome the limitations of the EPR effect, such as heterogeneity in vascular permeability and variable interstitial pressures that can impede nanoparticle penetration in certain tumors. To address this, techniques such as size modulation, surface charge optimization, and shape engineering are being explored to maximize the therapeutic efficacy of passively targeted nanoparticles [43,54,55].

The integration of passive targeting with other therapeutic strategies has also been of interest. For example, its combination with hyperthermia, where localized heat is used to temporarily enhance vascular permeability at the tumor site, has shown promise in increasing the accumulation of nanoparticles in tumors, thereby enhancing the therapeutic effect without increasing systemic side effects. This synergistic approach was effectively demonstrated in a study by Basu et al., where magnetic nanoparticles were combined with localized hyperthermia to enhance drug delivery to breast tumors [56]. The potential of passive targeting is also being expanded through technological innovations that allow for the real-time monitoring of nanoparticle distribution and tumor targeting efficacy. One such advancement is the development of imaging-capable nanoparticles, which can be tracked using various imaging modalities such as magnetic resonance imaging (MRI), PET, or fluorescence imaging. This dual functionality not only aids in confirming the accumulation of nanoparticles at the tumor site but also facilitates the optimization of treatment regimens in a patient-specific manner. The integration of diagnostic and therapeutic functions—termed theranostics—has become an exciting area of research, offering a streamlined approach to personalized cancer therapy. A good example is the study by Chauhan et al. on magnetic nanoparticles used for MRI and hyperthermia, which highlighted their use in providing real-time feedback on treatment efficacy and enhanced drug delivery through the EPR effect [57]. The combination of nanotechnology with genetic engineering presents another very promising strategy in cancer treatment. For example, gene therapy, when combined with nanoparticle delivery systems, can precisely target cancer cells while sparing healthy tissues. Recent research by Li et al. explored the use of lipid-based nanoparticles for the delivery of CRISPR-Cas9 systems targeting specific oncogenes in breast cancer cells. Their findings suggest that such approaches could potentially lead to highly effective and less-invasive alternatives to traditional chemo- and radiotherapy, offering a new paradigm in cancer treatment [58]. Additionally, combination therapies that utilize nanoparticles to deliver multiple therapeutic agents simultaneously are showing great potential [59]. These approaches can synergistically enhance treatment efficacy and overcome drug resistance. For example, studies have demonstrated that using nanoparticles to co-deliver chemotherapeutics and immunomodulatory agents can significantly improve therapeutic outcomes [59,60]. The quest for optimizing nanoparticle delivery also extends to enhancing the stability and circulation time of nanoparticles in the bloodstream. Innovations in nanoparticle coating materials have shown promising results in that regard. A recent study by McMullen focused on the development of a new zwitterionic coating for nanoparticles that significantly reduced the uptake by mononuclear phagocyte systems, thus prolonging the systemic circulation time. This advancement could lead to higher drug concentrations being delivered to the tumor site, thereby improving the therapeutic index of anticancer drugs [61].

### 4.2. Active Targeting

Active targeting in nanoparticle-mediated drug delivery for cancer involves the use of molecules that specifically recognize and bind to receptors or biomarkers overexpressed on the surface of cancer cells. This approach aims to increase the specificity and efficacy of drug delivery to cancer tissues.

**Figure 2:**
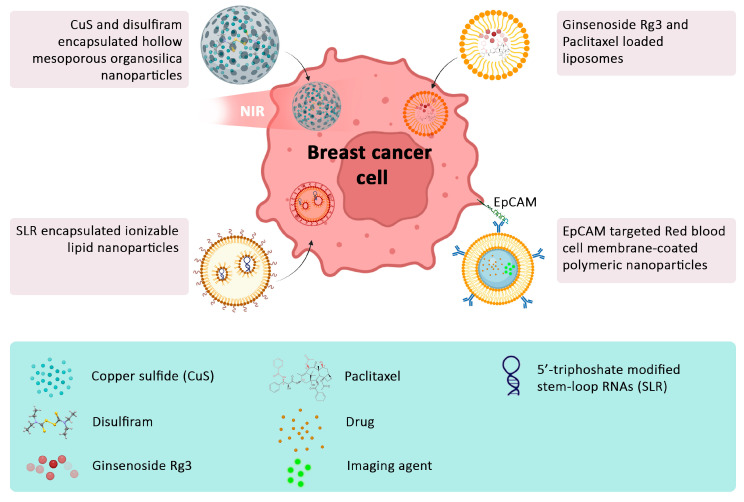
Diagram of selected nanoparticle-based strategies for breast cancer treatment. This diagram showcases various innovative nanoparticle formulations aimed at targeted treatment and imaging of breast cancer. It highlights the use of mesoporous nanoparticles, ionizable lipid nanoparticles, and targeted liposomes, each designed to optimize drug delivery, minimize toxicity, and enhance diagnostic accuracy. NIR: near-infrared radiation, EpCAM: epithelial cell adhesion molecule.

For example, by using antibodies or peptides that can bind to cancer-associated transmembrane receptors, surface-modified nanoparticles can specifically target cancer cells, increasing the efficacy of cancer treatments while reducing side effects [6,7,41]. In addition, nanoparticles can be designed to release drugs in a controlled manner over time, which can reduce the frequency of dosing (Figure 2) [62]. Smart nanomedicines can also cross biological barriers (such as the blood–brain barrier) and reach specific sites in the body to enhance the loaded drug’s potency [62].

Recent advancements have shown the potential of antibody fragments, such as Fab, Fab’, Fv, single-chain variable fragments (ScFv), and single-domain antibodies (nanobodies), as targeting ligands for nanoparticles [63]. These fragments are characterized by their highly specific targeting ability, cost-effective production, and genetic manipulability. Their smaller molecular size enhances tissue penetration and allows for easy conjugation into multifunctional delivery systems [64]. This conjugation significantly improved therapeutic efficacy [65].

Moreover, the integration of antibody fragments into nanoparticle systems has shown promising results in enhancing the specificity and efficacy of drug delivery. These conjugates have already exhibited their specific targeting ability and enhanced cytotoxicity in pancreatic cancer cells, highlighting their potential application in breast cancer therapy [63,64].

A pivotal study by Marshall et al. exemplifies this innovation through the development of biomimetic targeted theragnostic nanoparticles for breast cancer treatment [19]. Utilizing the principles of biomimetics, the study used nanoparticles cloaked in human red blood cell membranes, modified with targeting ligands (Figure 2). These nanoparticles encapsulated both chemotherapeutic agents and imaging markers and exhibited an enhanced ability to target epithelial cell adhesion molecule (EpCAM)-positive MCF-7 breast cancer cells [19]. The dual functionality not only facilitated targeted drug delivery but also enabled precise imaging of cancer cells, leveraging the unique advantages of nanoscale engineering to overcome the limitations of conventional treatments. This exemplifies a leap towards theranostics—a blend of therapy and diagnostics [66], and it also signifies a shift towards personalized medicine by monitoring cancer burden, which can be translated into adjustments of treatment regimens, to optimize outcomes. This example also underscores the potential of nanotechnology in facilitating a multifaceted approach to cancer treatment and detection.

The work of Ying Zhu et al. explored an equally compelling approach where lipid-based nanoparticles were used to combine the merits of targeted drug delivery with immunotherapy [67]. They encapsulated ginsenoside Rg3, a ginseng saponin that has immunomodulatory activity, and paclitaxel within liposomes, employing a dual-targeting mechanism to overcome the tumor microenvironment’s protective barriers and directly attack cancer cells (Figure 2) [67]. Merging targeted delivery with cytotoxic and immunotherapeutic payloads signifies a substantial advancement over conventional therapy, offering new treatment options for patients with drug-resistant breast cancer. Recent innovations in this approach include the use of nanoparticles specifically targeting tumor entities. A pivotal advancement in this field involved the use of ligands such as antibodies, aptamers, or small molecules with the ability to bind to cancer-specific receptors. For instance, the HER2 receptor is overexpressed in approximately 30% of breast cancer cases and has been effectively targeted using modified nanoparticles [68,69]. Lastly, the exploration of multi-functional nanoparticles that combine targeted drug delivery with other treatment modalities, such as hyperthermia or radiotherapy, is opening new avenues for comprehensive cancer treatment strategies. An innovative example by Herea et al. involved magnetic nanoparticles that were guided to the tumor site by external magnets and induced localized hyperthermia when exposed to alternating magnetic fields. This treatment modality disrupted tumor cells and enhanced the uptake of chemotherapeutic drugs, significantly improving treatment outcomes [70].

Recent advancements in nanotechnology have significantly improved the treatment strategies for bone metastasis in breast cancer. Nanoparticles offer unique advantages in delivering therapeutic agents directly to bone metastatic sites, enhancing drug bioavailability and reducing systemic toxicity [71]. Various types of nanoparticles, such as polymeric micelles, liposomes, and dendrimers, have been developed to target bone metastases effectively. These nanoparticles can be functionalized with ligands that have high affinity for bone tissues, such as alendronate, which ensures the selective delivery of drugs to the bone microenvironment [71].

In addition to their targeting capabilities, nanoparticles can improve the immunosuppressive tumor microenvironment and activate tumor-killing T-cells, thereby enhancing the therapeutic effect while minimizing adverse effects on normal tissues. Studies have demonstrated the efficacy of nanoparticles in carrying therapeutic agents, such as bisphosphonates and chemotherapeutic drugs, which not only target tumor cells but also inhibit osteoclast-mediated bone resorption [72,73]. This dual action is particularly beneficial in treating osteolytic bone metastases, which are characterized by excessive bone degradation [73].

Additionally, nanotechnology has shown promise in the targeting triple-negative breast cancer as nanocarriers. Triple-negative breast cancer (TNBC), comprising around 20% of all breast cancers, presents significant challenges in treatment due to the absence of estrogen, progesterone, and HER2 receptors, rendering it unresponsive to conventional targeted therapies [74]. This subtype is known for its aggressive behavior, high recurrence rates, and resistance to chemotherapy, largely attributed to breast cancer stem cells (BCSCs) which possess self-renewal, differentiation, and tumorigenic potential [74].

Recent advances in nanotechnology have provided promising strategies for targeting BCSCs and improving treatment outcomes for TNBC patients. Nanocarriers, designed to deliver therapeutic agents directly to BCSCs, offer several advantages such as enhanced drug bioavailability, stability, and site-specific delivery while minimizing off-target effects. These carriers include polymeric nanoparticles, liposomes, dendrimers, and carbon nanomaterials [75].

Nanocarrier-based therapies have shown potential in targeting specific molecular pathways and cellular processes critical for BCSC survival. For instance, polymeric nanoparticles can be engineered to deliver drugs like doxorubicin directly to BCSCs, enhancing therapeutic efficacy while reducing systemic toxicity. Similarly, liposomes and dendrimers have been utilized to encapsulate chemotherapeutic agents, providing controlled release and improved the targeting of BCSCs [76].

Moreover, targeting surface markers such as CD44 and CD133 and key signaling pathways like Notch, Wnt, and Hedgehog have been explored using nanoparticle-based systems. These approaches aim to eliminate BCSCs or promote their differentiation into non-tumorigenic cells, thereby enhancing their susceptibility to conventional therapies [76].

One of the innovative strategies involves using F3 peptide-targeted liposomes which have demonstrated a remarkable ability to selectively deliver therapeutic agents to TNBC cells, particularly targeting the cancer stem cells responsible for tumor growth and recurrence [75,77]. This targeted approach holds great promise for improving TNBC treatment outcomes by addressing the challenges of tumor heterogeneity and drug resistance [77].

Another promising avenue is the use of dendrimers as nanocarriers. Polyamidoamine (PAMAM) dendrimers, for instance, have been studied for their ability to deliver anti-cancer drugs specifically to TNBC cells, enhancing drug potency and minimizing off-target effects. These dendrimers can be functionalized with ligands to improve targeting capabilities and facilitate the efficient delivery of therapeutic agents to cancer cells [75,78].

In summary, numerous cutting-edge nanoparticle innovations harbor the potential to fundamentally transform the landscape of breast cancer therapy, moving towards more personalized, effective, and less-invasive treatment options. As this field continues to evolve, it holds the promise of significantly improving patient outcomes by reducing side effects and enhancing the specificity and efficacy of cancer treatments.

### 4.3. Responsive Nanoparticles

Nanoparticle-based phototherapy offers additional novel therapeutic approaches against breast cancer. Photodynamic therapies (PDT) and PTT employ light of varying wavelengths to induce photochemical or photothermal changes within target tissues [79]. In PDT, nanoparticles serve as carriers for photosensitizing agents, facilitating the generation of reactive oxygen species upon light activation. Conversely, PTT leverages the nanoparticles’ ability to absorb and convert light into heat, effectively destroying cancer cells through localized hyperthermia [80]. Both techniques hold immense promise for refining breast cancer treatments, offering enhanced specificity and curative efficacy [80].

Zhang et al.’s ground-breaking work with hollow mesoporous organosilica nanoparticles represents a major leap forward in PTT [81]. Their meticulously engineered nanoparticles encapsulate copper sulfide (CuS) for its photothermal properties, paired with the chemotherapeutic agent disulfiram (DSF). This combination facilitated a twofold approach against tumors: near-infrared light-triggered photothermal activation causing localized hyperthermia and the concurrent release of DSF, which directly impacted cancer cell viability (Figure 2) [81]. Such an approach exemplifies the precision and localized action of nanoparticle-mediated treatments, aiming to maximize tumor destruction while sparing surrounding healthy tissue [81].

A second novel approach using ionizable lipid nanoparticles for delivering 5′ triphosphate-modified stem-loop RNAs (SLRs) has also shown promise in breast cancer therapy (Figure 2) [82]. Using these SLRs as a nanoparticle payload, they activated the retinoic acid-inducible gene I (RIG-I) pathway, thus enhancing tumor immunogenicity and response to immune checkpoint inhibitors. Interestingly, these SLRs effectively activated RIG-I within the tumor microenvironment and inhibited tumor growth when tested in vivo using melanoma and breast cancer mouse models. Notably, systemic SLR-loaded nanoparticle administration enhanced the infiltration of CD8^+^ and CD4^+^ T-cells into breast cancer tissue, enhancing the effects of administered antibodies against programmed cell death protein-1, generally known as PD-1.

Early detection is crucial as it often allows for more treatment options and a better chance of successful disease management. In this context, the unique optical properties of gold nanorod particles allow them to absorb and scatter light in a way that can be used for imaging and treatment [21]. Black phosphorus, on the other hand, is a material that is sensitive to changes in the environment, which can help in sensing the presence of cancer biomarkers. The sensor created by Liang et al. is highly sensitive and capable of detecting and targeting breast cancer biomarkers—molecules that indicate the presence of cancer—at much earlier stages than many current diagnostic methods [37].

Once these biomarkers are detected, the same nanoparticles can then play a role in therapy, potentially using techniques like those developed by Zhang et al., where they target the cancer cells with a localized treatment, minimizing damage to healthy tissue [37,81].

As the field progresses, it is crucial to address whether advances in nanoparticle applications are equally promising across different breast cancer subtypes and at various stages of the disease, including early and metastatic stages. This understanding is essential for translating these innovations into personalized medicine [83]. By tailoring nanoparticle-based therapies to the specific molecular and clinical characteristics of each patient’s cancer, clinicians can enhance treatment efficacy and reduce adverse effects. Moreover, continued research into combination therapies and the integration of multi-omics approaches will likely propel the field forward, offering new avenues for personalized and effective cancer treatment strategies.

## 5. Challenges, Translation, and Future Directions of Nanoparticles in Clinical Breast Cancer Treatment

Despite their tremendous potential, nanoparticle-based treatments are facing several safety challenges. Unlike traditional drugs, nanoparticles have unique interactions with biological systems, raising concerns about long-term biodistribution, potential toxicity, and unforeseen immune responses [84]. Studies have demonstrated that the nanoscale size of these particles enables them to interact with biological membranes and intracellular components in complex ways, potentially leading to oxidative stress, inflammation, and even genotoxic effects [85,86]. For example, recent in vivo work by Ernst et al. utilized bioluminescence tracking to monitor the distribution of nanoparticles post-administration, revealing preferential accumulation in the liver and spleen for extended periods of over 100 days, raising questions about long-term implications for organ function and health [87].

Furthermore, the clinical translation of nanoparticles is hindered by manufacturing and regulatory hurdles. Meeting clinical-grade standards necessitates not only innovation in manufacturing practices but also adherence to stringent good manufacturing practice (GMP) standards [88]. Evolving techniques, such as microfluidics and continuous flow synthesis, are pivotal in enhancing the reproducibility and preservation of the therapeutic properties of nanoparticles [89,90,91]. This task is further complicated by a regulatory framework that often lacks specific guidance for the unique attributes and actions of nanoparticles [92]. A comprehensive review by the International Nanosafety Consortium (2024) emphasizes the urgent need for standardized nanoparticle characterization protocols to inform regulatory decisions [93].

The cost of developing and deploying nanoparticle-based therapies cannot be overlooked, as the investment in high-tech manufacturing processes and compliance with regulatory standards invariably inflates expenses [92]. Economic evaluations, such as those conducted by Milewska et al., have revealed that the production cost of nanoparticle formulations can be up to tenfold higher than that of conventional therapeutics, a factor that can significantly restrict patient access [94]. This is further compounded by healthcare disparities, where advanced treatments remain beyond the reach of underprivileged populations.

Addressing the dual challenges of safety and cost requires an integrated approach that spans research, regulatory, and policy-making spheres. Enhanced safety evaluations, incorporating advanced in vitro and in vivo models that mimic human biological systems more closely, can provide deeper insights into nanoparticle behavior and interactions. This, combined with ongoing monitoring in clinical settings, can help to identify and mitigate potential adverse effects more effectively. On the economic front, fostering collaborations between the public and private sectors can facilitate investment in the research and development of nanoparticle therapeutics. Regulatory bodies can also play a pivotal role by streamlining approval processes and establishing clear guidelines for nanoparticle therapeutics, potentially reducing development costs and timelines.

As the field progresses, it is crucial to address whether advances in nanoparticle applications are equally promising across different breast cancer subtypes and at various stages of the disease, including early and metastatic stages. This understanding is essential for translating these innovations into personalized medicines. By tailoring nanoparticle-based therapies to the specific molecular and clinical characteristics of each patient’s cancer, clinicians can enhance treatment efficacy and reduce adverse effects. Moreover, continued research into combination therapies and the integration of multi-omics approaches will likely propel the field forward, offering new avenues for personalized and effective cancer treatment strategies.

## 6. Conclusions

The emergence of nanoparticles has led to a new era of breast cancer diagnosis and treatment, providing more targeted, effective, and personalized therapeutic strategies. The unique properties of nanoparticles, including their small size and modifiable surface chemistry, offer immense potential for overcoming the challenges associated with traditional treatment modalities. It is evident that nanoparticles hold promise across various applications in breast cancer management, including imaging, targeted therapy, drug delivery, and immunotherapy. Studies have demonstrated the effectiveness of nanoparticle-based approaches in improving the early detection of metastasis, enhancing drug delivery to cancer cells, overcoming multidrug resistance, and stimulating antitumor immune responses.

Despite significant progress, the clinical adoption of nanoparticle-based therapies faces challenges, including chemical feasibility, biodistribution, efficacy, safety, scalability, and regulatory hurdles [29,95]. Future directions should focus on addressing these barriers, with particular attention to how multi-omics approaches and combination therapies can enhance the specificity and efficacy of treatments and how these findings integrate into personalized medicine frameworks. The use of novel approaches, such as the integration of multi-omics data into nanoparticle-based therapies and the development of combination therapies, will likely propel the field forward, offering more personalized and effective treatment options for breast cancer patients.

The translation of nanoparticle-based therapies from preclinical studies to clinical practice requires rigorous testing, optimization of the manufacturing processes, and navigation through regulatory approval pathways. Addressing these challenges in parallel with technological development will be essential to ensure the safe and effective integration of nanoparticle-based therapies into routine clinical care for breast cancer patients.

Overall, nanoparticle-based therapies are showing very promising therapeutic effects and herald a significant shift towards personalized and less-invasive treatment strategies, potentially improving the survival rates of breast cancer patients. The incorporation of such technologies into clinical practice, however, will demand a holistic view of their impact, considering therapeutic benefits alongside ethical, economic, and accessibility issues.

## Figures and Tables

**Table 1:** Composition and physicochemical characteristics of nanoparticles in breast cancer research.

Nanoparticle Type	Surface Modification	Size Range (Biodegradability)	Targeting Mechanism
Lipid-based [9,10] (e.g., cholesterol, phospholipids)	PEGylation, targeting ligands (e.g., antibodies)	30–100 nm (yes)	Passive (EPR effect), active (ligand–receptor interaction)
Polymeric [11,12] PLGA, PEG	Targeting ligands, therapeutic agents	10–100 nm (yes)	Passive (EPR effect), active (ligand–receptor interaction)
Inorganic [13,14] gold, iron oxide	Silica coating, targeting moieties	2–100 nm (inconsistent)	Magnetic targeting, photothermal
Protein-based [15,16], albumin, ferritin	Natural ligands (e.g., folic acid)	10–100 nm (inconsistent)	Active (natural ligand–receptor interaction)
Carbon-based [17], carbon nanotubes, graphene	Functional groups (e.g., -COOH, -NH_2_)	1–100 nm (inconsistent)	Passive accumulation, active (functionalization)
Hybrid [18], combination of above materials	Combination of above modifications	Depends (inconsistent)	Combines multiple targeting mechanisms

EPR: Enhanced permeation and retention, PEG: polyethylene glycol, PLGA: poly(lactic-co-glycolic acid).

**Table 2:** Diagnostic tools in clinical trials (ClinicalTrials.gov, 28 April 2024).

Nanoparticle Composition	Intervention	Clinical Trial Number (Phase)
SPIO	Delayed sentinel lymph node dissection (d-SLND)	NCT04722692 (III)
	Sentinel lymph node detection (SLND)	NCT06169072 (II) NCT05359783 (I/II) NCT05985551 (NA)
SPIO (MagTrace and Magseed)	Axillary lymph node identification before neoadjuvant chemotherapy	NCT05625698 (NA) NCT06104371 (NA)
	Tracer in sentinel node biopsy in breast cancer	NCT05161507 (NA)
Carbon nanoparticle suspension	Ultrasound-assisted tracer-guided sentinel lymph node biopsy	NCT04951245 (III)
	Location and biopsy of axillary lymph nodes	NCT04482803 (NA)
	Axillary lymph node identification before neoadjuvant chemotherapy	NCT03355261 (NA)
	Stained region lymph node biopsy. Regional lymph node radiotherapy.	NCT05939830 (NA)
Quantum dots	Bioimaging with somatostatin analog (Veldreotide)-coated quantum dots	NCT04138342 (I)

SPIO: superparamagnetic iron oxide nanoparticle, NCT: The National Clinical Trial number.

**Table 3:** Breast cancer nanoparticle medicines currently used in clinic or under development.

Drug	Nanoparticle Composition (Properties)	Phase (Clinical Trial Number)
Abraxane	Albumin-bound paclitaxel (passive targeting via EPR)	Approved (abraxane.com)
Caelyx	Pegylated liposome doxorubicin	Approved
Genexol-PM	Polymeric micelle paclitaxel	Approved
Myocet	Liposome doxorubicin	Approved
Nanoxel M	Polymeric micelle paclitaxel	Approved
BIND-014	Accurin polymeric nanoparticles with docetaxel for injectable suspension	I (NCT01300533)
CALAA-01	Anti-ribonucleotide reductase M2 siRNA in AD-PEG-Tf (transferrin-targeting agent)	I (NCT00689065)
ELU001	C-Dot nanoparticle (FOLR1 targeting)	I/II (NCT05001282)
IMX-110	Poly-kinase inhibitor and doxorubicin in PEG-PE-based nanoparticle	I/II (NCT03382340)
IPI-549 (TNBC)	Pegylated liposomal doxorubicin	I (NCT03719326)
mRNA-2752	Lipid nanoparticle (OX40L, IL-23, and IL-36γ mRNA)	I (NCT03739931)
MT-302	mRNA lipid nanoparticles (chimeric antigen receptor expression in myeloid cells, targeting TROP2-expressing cancer cells)	I (NCT05969041)
NK105	Micellar nanoparticle paclitaxel	III (NCT01644890)
Rexin-G	Retroviral	I/II (NCT00505271
SNB-101	SN-38 nanoparticle formulation	I (NCT04640480)
X-ray irradiation	AGuIX gadolinium-based nanoparticles (Brain-directed stereotactic radiation)	II (NCT04899908)

TNBC: triple-negative breast cancer, PEG-PE: polyethylene glycol–phosphatidylethanolamine conjugates, AD-PEG-Tf: adamantane-PEG-transferrin.

## Data Availability

No new data were created or analyzed in this study. Data sharing is not applicable to this article.
